# Restoration of Over-Ground Walking via Non-Invasive Neuromodulation Therapy: A Single-Case Study

**DOI:** 10.3390/jcm12237362

**Published:** 2023-11-28

**Authors:** Monzurul Alam, Yan To Ling, Md Akhlasur Rahman, Arnold Yu Lok Wong, Hui Zhong, V. Reggie Edgerton, Yong-Ping Zheng

**Affiliations:** 1 Department of Biomedical Engineering, The Hong Kong Polytechnic University, Hong Kong, China; jane.yt.ling@connect.polyu.hk (Y.T.L.); akhlas.physio@gmail.com (M.A.R.); yongping.zheng@polyu.edu.hk (Y.-P.Z.); 2 Centre for Developmental Neurobiology, Institute of Psychiatry, Psychology and Neuroscience, King’s College London, London SE1 9NH, UK; 3 Department of Physiotherapy, Centre for the Rehabilitation of the Paralysed, Savar Union 1343, Bangladesh; 4 Department of Rehabilitation Sciences, The Hong Kong Polytechnic University, Hong Kong, China; arnold.wong@polyu.edu.hk; 5 Rancho Research Institute, Rancho Los Amigos National Rehabilitation Center, Downey, CA 90242, USA; zhonghui731@gmail.com (H.Z.); vre@ucla.edu (V.R.E.); 6 Neurorestoration Center, University of Southern California, Los Angeles, CA 90089, USA; 7 Department of Neurological Surgery, Keck School of Medicine, University of Southern California, Los Angeles, CA 90089, USA

**Keywords:** chronic spinal cord injury, sensorimotor rehabilitation, transcutaneous electrical stimulation, neuromodulation, over-ground walking

## Abstract

Spinal cord injuries (SCI) can result in sensory and motor dysfunctions, which were long considered permanent. Recent advancement in electrical neuromodulation has been proven to restore sensorimotor function in people with SCI. These stimulation protocols, however, were mostly invasive, expensive, and difficult to implement. In this study, transcutaneous electrical stimulation (tES) was used to restore over-ground walking of an individual with 21 years of chronic paralysis from a cervical SCI. After a total of 66 weeks of rehabilitation training with tES, which included standing, functional reaching, reclined sit-up, treadmill walking, and active biking, significant improvement in lower-limb volitional movements and overall light touch sensation were shown as measured by the International Standards for Neurological Classification of Spinal Cord Injury (ISNCSCI) score. By the end of the study, the participant could walk in a 4-m walking test with the aid of a walking frame and ankle–foot orthoses. The successful sensorimotor recovery of our study participant sheds light on the future of non-invasive neuromodulation treatment for SCI paralysis.

## 1. Introduction

A spinal cord injury (SCI) is an immediate, severe, disabling, and life-altering neurological impairment that has a significant adverse impact on patients, their families, and the healthcare system, as well as their physical, emotional, social, and occupational well-being [1,2,3]. Spinal cord injuries can cause severe damage to individuals, often leading to paralysis as they lose conscious control below the damaged area. SCI substantially impacts an individual’s bodily functions, resulting in loss of sensation and limb control and poor bladder and bowel control [4,5]. Secondary issues include autonomic dysreflexia, cardiovascular disease, osteoporosis, spasticity, infections, and pressure ulcers [6]. Chronic pain is another common complication after SCI, affecting up to 80% of individuals with SCI [7]. It can be due to nerve damage, musculoskeletal imbalances, or psychological factors that worsen physical function and lower the quality of life for people with SCI [2]. People with SCI have been found to have a higher level of prevalence of depression and anxiety, ranging from 22% to 40% [8]. Emotional consequences after SCI have been widely reported; for example, 30% of individuals with SCI have a risk of developing a depressive disorder in the rehabilitation phase, and 27% of them show depression symptoms when living in the community [9]. It has been reported that the re-employment rate ranges from 14% to 44%, and social involvement in terms of an individual’s social roles and interactions varies due to several factors, including the characteristics of the patient and the definition of employment [10]. Physical barriers can prevent people with SCI from participating in social activities and community events. Furthermore, SCI can limit a person’s ability to work, which can have significant economic and social consequences. Unemployment and underemployment are common among people with SCI, which can affect their financial stability and social status [11]. Given the significant negative effects of SCI on individuals with SCI, caregivers, the healthcare system, and society, reversing paralysis in people with SCI is one of the highest priorities in treating these individuals.

Paralysis was long thought to be irreversible, but recent advances in spinal cord neuromodulation therapies have shown remarkable success in restoring movements and sensation to the paralyzed areas [12,13]. These successes have been achieved using an invasive method called epidural electrical stimulation, in which electrodes are implanted in the dura mater of the spinal cord. Although successful, the surgery involved in this treatment is expensive and can cause various complications and risks [14]. Non-invasive spinal cord neuromodulation, specifically transcutaneous electrical stimulation (tsDCS and tsPCS), uses transcutaneous electrical stimulation through the spinal cord and its peripheral nerves and is an effective treatment protocol for SCI and other neurological conditions. Successful applications have been demonstrated in the conditions of chronic pain [15], spasticity [16], respiratory problems [17], cardiovascular ischemia [18], neuropathic bladder [19], bowel dysfunction [20], and upper and lower limb function including fine motor function with digit function [21,22]. The impact of the possibility of regaining mobility could become even greater, realizing that the intervention can be developed for home use and is less challenging technologically and economically. Further reports amplify the impact of the non-invasive strategy used in this study [23,24].

In a previous report [25], we described how a chronic SCI individual with tetraplegia, who had been wheelchair-bound for the previous 21 years following a traumatic cervical cord injury in a car accident, regained volitional movements in bilateral leg muscles after 16 weeks of non-invasive spinal cord stimulation (tES) and activity-based physical training. The current report reveals the effects of an additional 44 weeks of tES and progressive physical training in helping the study participant walk independently.

## 2. Materials and Methods

### 2.1. Study Participant

Our study participant was a 48-year-old woman who was involved in a road traffic accident 21 years ago (cervical burst fracture) and sustained a C7 cervical cord injury resulting in Brown Séquard Syndrome. Although her bilateral distal upper limb muscles remained impaired and she could not fully flex her fingers, she was able to perform most of her upper limb tasks for moderate-intensity household and functional activities. Further, she had moderate trunk control, allowing her to sit on the edge of a bed with the support of her hands. However, her lower limb functions were severely limited following the injury. As a result, she had been wheelchair-bound since her injury. At the beginning of the study, she had very limited active movement in the right leg, while her left leg was completely paralyzed. In addition, moderate muscle spasm was noted in both legs, especially in the left leg. Some weak and altered sensation was retained in her saddle region, but no motor function was preserved in the bowel/bladder.

### 2.2. Stimulation Protocol

**Figure 1:**
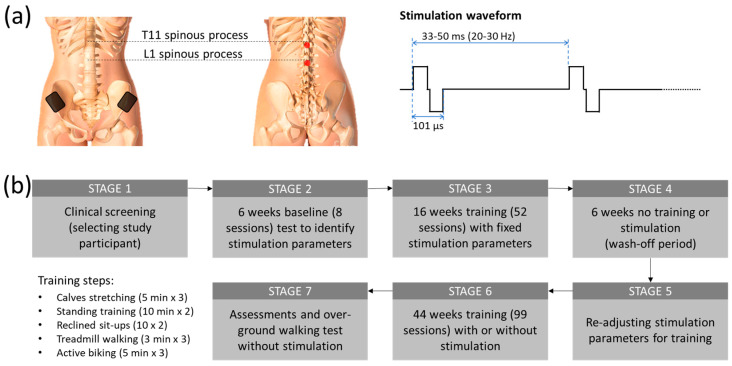
(**a**) Overall training methods and study procedures. After passing the clinical screening (STAGE 1), eight transcutaneous electrical stimulation (tES) sessions were conducted on the study participant on assisted standing and walking to determine the optimum stimulation parameters for training, followed by pre-training assessments (STAGE 2). A total of 16 weeks of physical training were then provided 3 times/week in conjunction with stimulation (tES). The training steps included a total of 15 min stretching of calves; 2 sets (7–10 times each) of unassisted sit-ups on a reclined position; 20, 15, and 9 min of assisted standing, biking, and treadmill walking. After 52 intensive training sessions (STAGE 3), functional reassessments were conducted to determine the participant’s improvement. Following this, a 6-week break from the stimulation training was provided to examine the retainment of the participant’s newly gained functional abilities (STAGE 4). Following the break, the stimulation parameters were re-adjusted for the new functionality of the participant (STAGE 5). Then, 44 weeks of discontinuous training with and without stimulation were provided to see the effect on motor learning (STAGE 6). Upon completion, final post-training assessments, including, for the first time, an overground walking test, were conducted (STAGE 7). (**b**) tES setup. (Left) Stimulation electrode placements in both anterior and posterior views. Two 3.2 cm diameter electrodes were placed at the midline, immediately below the T11 and L1 spinous processes. Two internally connected 6 × 9 cm^2^ rectangular electrodes were also attached to the skin above the iliac crests. (Right) Stimulation waveform of biphasic pulses (50 µs positive and 50 µs negative pulse currents with 1 µs inter-pulse interval) delivered at 20–30 Hz stimulation frequency.

Two constant current stimulators (Model DS8R, Digitimer, UK) were utilized in this study to simultaneously stimulate the participant’s T11 and L1 spinal segments (Figure 1a). An arbitrary function generator (Model AFG1022, Tektronix, Inc., Beaverton, OR, USA) was used to generate a 9.4 kHz burst trigger at 20 to 30 Hz. From this trigger, each stimulator produced a biphasic tES (50 µs negative and 50 µs positive pulse currents with 1 µs inter-pulse interval). Two 3.2 cm diameter self-adhesive electrodes (ValuTrode, Axelgaard Manufacturing Co., Ltd., Fallbrook, CA, USA) were placed at the midline, immediately below the T11 and L1 spinous processes, to deliver tES currents at an intensity ranging from 20 mA to 120 mA. Two internally connected 6 × 9 cm^2^ self-adhesive rectangular electrodes (Guangzhou Jetta Electronic Medical Device Manufacturing Co., Ltd., Guangzhou, China) were also attached to the skin above the iliac crests to act as a sink for the stimulation current.

### 2.3. Study Procedure

After completing an initial screening for the subject’s cardiac health for physical activity and a bone density evaluation by an independent physician, the participant was included in a non-invasive spinal cord stimulation called transcutaneous electrical stimulation (tES) trial. Figure 1b shows the overall outline of the study procedure. In a previous report [25], we reported the first part (STAGE 1–STAGE 4) of the study, where the study participant regained volitional leg movements and weight-bearing standing after 16 weeks of stimulation and physical training. In brief, after the baseline sensorimotor assessment using the International Standards for Neurological Classification of Spinal Cord Injury (ISNCSCI), the participant attended eight baseline sessions for pre-training tests to identify the optimal tES parameters from lower limb motor evoked potentials for the physical training. The study participant subsequently attended 16 weeks of 2-h training sessions at an average of 3 times a week (the subject missed some sessions due to her schedule but attended at least 2 times per week. We also compensated for the missed sessions with additional sessions to maintain the average training constant). Each training session started with a set of three 5-min stretches (a total of 15 min), with a 2-min rest between sets. Each tES-assisted physical training session was divided into four parts in the following order: (1) a set of three 10-min standing and functional reaching (a total of 30 min), with a 3-min break between sets; (2) a set of two 7–10 sit-ups on a reclined chair; (3) a set of three 3 min of treadmill walking with 20–30% body-weight support (a total of 9 min) with a 3-min break between sets; and (4) a set of three 5-min forward and reverse biking (a total of 15 min), with a 3-min break interspersed between sets. Blood pressure was regularly checked between each training set. During the standing and functional reaching part, the participant was instructed to perform some trunk and lower body exercises, such as side and forward bending and/or squatting, depending on the physical condition on a given treatment date. The period of walking on the treadmill and load of biking exercise was increased gradually based on the participant’s condition as the training progressed. Sometimes, the subject could not reach the expected training duration because of fatigue and other factors. The reduced duration was, however, normally within 30% of the expected. A final neurological assessment (ISNCSCI) was conducted at the end of the study to evaluate the post-treatment effect.

### 2.4. Standing and Functional Reaching Training with tES

Optimum tES was chosen based on the participant’s comfort and reported ease of standing with the least physical support after multiple iterations over the first 6 weeks of the trial. The participant could sense the stimulation and provided verbal feedback during the process. The participant was unblinded to the parameter changes, and when the chosen tES parameters reached the optimum stimulation, the participant reported that she felt “more connected” to her paretic body parts; these parameters were used for stimulation for her standing training. The tES was delivered at 20 Hz at an intensity of 105 mA at the T11 level and 100 mA at the L1 level. Throughout the training period, the stimulation parameters were kept constant. Only the intensity was adjusted (±10 mA) based on the participant’s comfort. At the beginning of the training, manual support was given to the pelvis, knees, and feet. These supports were gradually lifted as the standing ability improved. After several initial training sessions, the participant was instructed to semi-squat from standing once she gained control over her knees. The same tES parameters were applied for the semi-squat training.

### 2.5. Reclined Sit-Ups with tES

After each standing session, our study participant was seated in a reclined wheelchair and completed 7 to 10 sit-ups. The same standing training tES parameters were used for the sit-ups exercise. Only the current intensity was adjusted to provide ease to do the task. The researcher verbally encouraged the study participant to complete all 10 sit-ups. An occasional break was given to allow her to catch breaths as needed. We increased the wheelchair backrest angle for the sit-ups once the study participant was able to complete all 10 sit-ups easily and regularly using the resistance training progression principle [26]. We evaluated the immediate response after the adjustment to ensure that the exercise would overload the target muscles without causing damage.

### 2.6. Treadmill Walking Training with tES

The tES was used to help our participant during body weight-assisted treadmill walking. During treadmill walking, two trainers assisted the participant’s legs to move throughout the gait cycle (1.125 km/h), while another trainer emulated pelvis rotation. Optimum stimulation parameters were chosen by the participant after multiple sessions throughout the first 6 weeks of the trial, using the same technique mentioned in the standing training. The tES was delivered at 30 Hz at an intensity of 90–110 mA at T11 and L1 levels. During the training session, the participant walked for up to three sets of 3 min on a moving treadmill belt with 20–30% body-weight support (a total of 9 min). A 2-min break was given between each walking training session. The stimulation parameters were kept constant throughout the walk.

### 2.7. Active Biking Training with tES

The tES was used to help our participant during forward and reverse biking. A motorized bike with passive and active operation options (MOTOmed viva2, RECK-Technik GmbH and Co. KG, Betzenweiler, Germany) was set on 10 cycles/min for 5 min for each session of forward and reverse biking with a 3-min rest time between each session. The study participant was encouraged to pedal at a speed higher than the preset biking speed while tES was delivered to her T11 and L1 spinal segments at 25 Hz. Following the assisted active biking exercise, another 2 min of passive biking (1-min forward and 1-min reverse) was given to relax the lower limb muscles.

### 2.8. Testing of Over-Ground Walking with and without tES

In the current study, tES was used to help the study participant regain volitional control of her paretic legs to restore over-ground walking. The participant was encouraged to move her legs voluntarily. At the early stage of the training, the tES current was delivered during the volitional activities. However, our study participant was able to move her legs nearly without the assistance of tES at the end of the 16-week training. By the end of the study, our study participant could ambulate over-ground with the assistance of a walker even without the tES. A 4-m walking test was conducted to evaluate her recovery from walking. Body kinematics and lower limb muscle activities were captured using an integrated motion capture system (Vicon Nexus, Vicon Motion Systems Ltd., Oxford, UK) and an 8-channel wireless electromyography EMG acquisition system (Trigno Avanti, ADInstruments, Otago, New Zealand). For EMG recording, four pairs of EMG electrodes were placed at the bilateral quadriceps muscle belly, tibialis anterior, hamstrings, and gastrocnemius. The EMG signal was digitized and saved on a computer at a sample rate of 2 kS/s for offline analysis. Videos were also shot with a digital camera during walking and juxtaposed with the kinematic data.

### 2.9. Data Analysis and Statistics

Gait and muscle dynamics were analyzed offline from the motion markers and EMG signals of lower-limb movements using a customized MATLAB script (MathWorks Inc., Natick, MA, USA). The difference between left and right leg gait performances was determined using paired *t*-tests. Statistical software (GraphPad Software Inc., La Jolla, CA, USA) was used for all statistical analyses. The significant level was set at 0.05.

## 3. Results

### 3.1. Improvement in Sensory and Motor Functions

**Figure 2:**
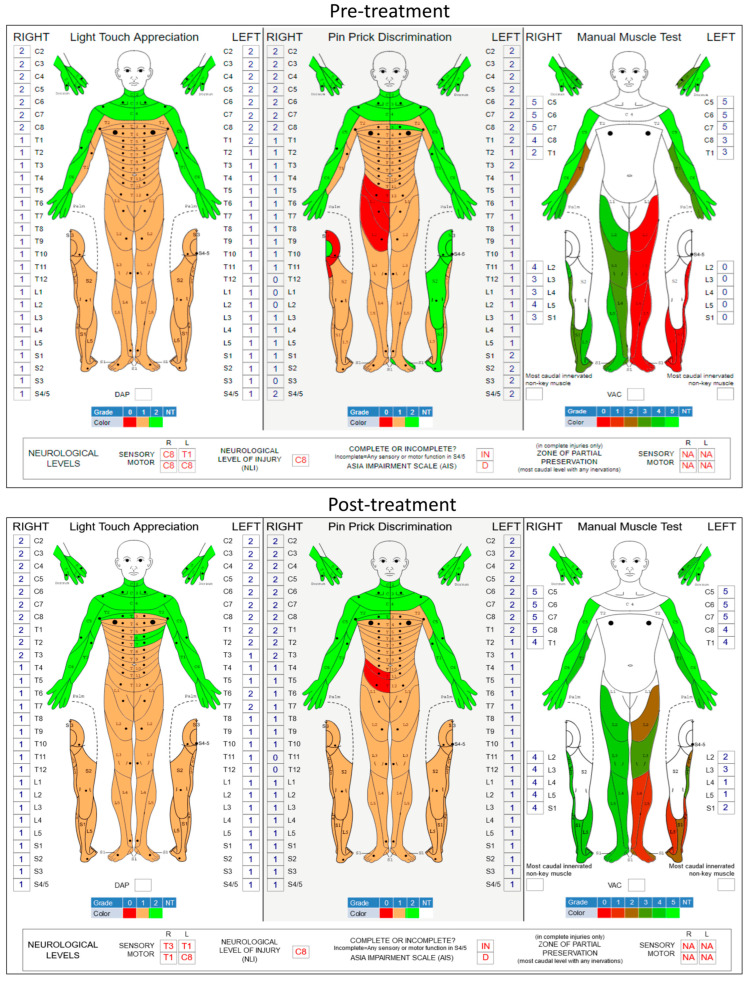
Pre- and post-training assessment of the participant. International Standards for Neurological Classification of Spinal Cord Injuries (ISNCSCI) worksheet scores before and after the treatment. Significant improvements in motor scores (manual muscle test) indicate the renversement of paralysis of the left lower limb. The total score of the five individual movements (hip flexion, knee extension, ankle dorsiflexion, long toe extension, and ankle plantar flexion) exhibited significant improvements (*p* = 0.009; two-tailed paired *t*-test) after stimulation treatment compared to the baseline. Significant changes in the light touch appreciation (*p* = 0.031, two-tailed paired *t*-test) indicate some sensory recovery, while the pin-prick sensations did not change significantly (*p* = 0.769, two-tailed paired *t*-test) compared to the baseline.

Figure 2 shows the pre- and post-treatment ISNCSCI scores. Improvement in both light touch appreciation and manual muscle tests is shown as the shift in color from red to green. The ISNCSCI score on left-leg volitional movements increased from a grade of 0 to a grade of 9 (*p* = 0.009; two-tailed paired *t*-test), while right-leg motor function improved from grade 17 to 20 (*p* = 0.071, two-tailed paired *t*-test). It has been observed in various spinal neuromodulation applications that stimulation enhances not just motor activity but also sensory function in SCI individuals. In the present study, the overall light touch sensation also significantly improved from grade 71 to grade 77 (*p* = 0.031, two-tailed paired *t*-test). However, pin-prick discrimination did not change (grade 73 to 72, *p* = 0.769, two-tailed paired *t*-test) after the tES therapy.

### 3.2. Restoration of Overground Walking Ability

**Figure 3:**
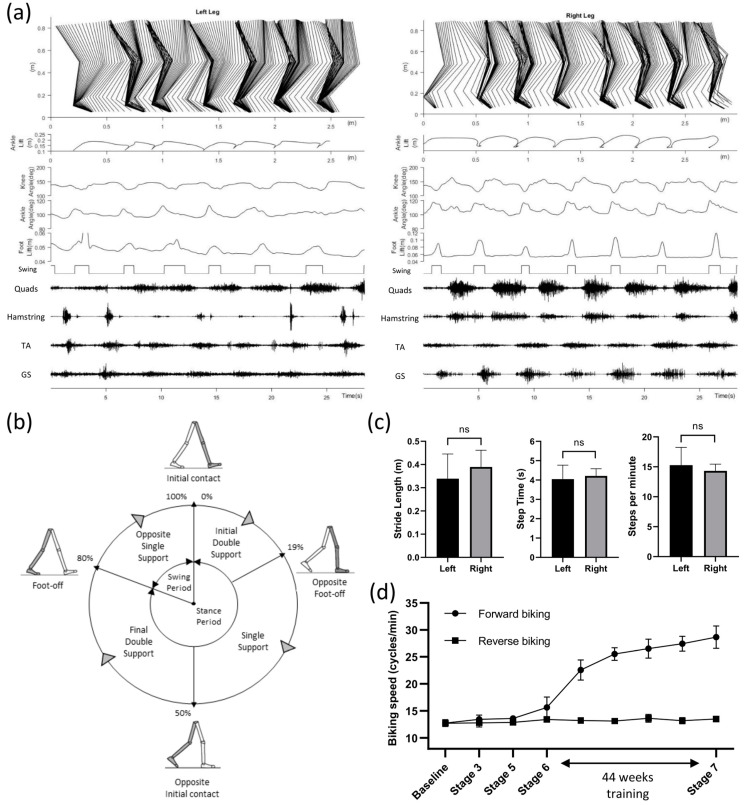
(**a**) Gait pattern and muscle dynamics of the left and right leg during over-ground walking with a walker. Stick diagram decomposition of lower-limb movements (1st row). The position of the ankle shows each stride (2nd row). Knee and ankle angles (3rd to 4th row). Foot lifting off the ground (5th row) is used to determine the stance and swing phase (6th row). Synchronized EMG signals showing activations of the Quadriceps, Hamstring, Tibialis Anterior (TA), and Gastrocnemius (GS) (7th to 10th row). (**b**) Normalized gait cycle of the right leg with 80% stance phase (19% initial double support, 31% single support, 30% final double support) and the rest 20% swing phase. (**c**) Average stride length, step period, and steps per minute (mean ± SD) for both legs. No significant difference was found between the left and right leg for all parameters (*p* = 0.318; *p* = 0.622; and *p* = 0.479; *t*-tests). (**d**) Average biking speed in forward and backward directions throughout different stages of the study. Significant improvement (*p* < 0.001; R2 value = 0.945; non-linear regression) in forward biking speed is observed during the 44-week discontinuous training period (STAGE 6).

At the end of the study, we tested the participant’s walking ability in a 4-m walking test with a high-speed motion capture system (Vicon Motion Systems Ltd., Oxford, UK) and a wireless EMG system (Trigno Avanti, ADInstruments, Dunedin, New Zealand). Due to long chronic paralysis, the study participant had shortened calf muscles and ankle invertors, which made her unable to place her heels on the ground while standing. To resolve this, we stretched her bilateral dorsiflexors. However, we did not see any significant improvements in the passive ranges of motion of her bilateral ankle eversion and plantarflexion. Hence, our study participant had to utilize ankle–foot orthoses (AFOs) to prevent excessive ankle inversion and plantarflexion during weight-bearing standing and walking. Figure 3 shows the gait pattern and muscle dynamics of the left and right leg during over-ground walking with a walker. Figure 3a shows details of motion and muscle activities, while Figure 3b,c summarizes the overall gait pattern and differences between the left and right legs. Although a clear stepping pattern can be observed, the participant often put her left foot even with the right foot, instead of ahead of the right, for each step (Figure 3a). Gait and muscle dynamics were further analyzed from the motion markers and EMG signals of lower-limb movements. The Stick diagram shows that the left leg had slower steps compared to the right leg, while the right leg had much smoother steps, as observed in the swing phases. This can be further observed in the foot, ankle, and knee position patterns (Figure 3a). Furthermore, the quadriceps and hamstring muscles showed more robust EMG signals on the right leg compared to the left leg. Figure 3b shows the normalized gait cycle of our study participant, illustrating symmetric phase relationships of temporal events and periods.

The gait cycle of the right leg had 80% stance phase (19% initial double support, 31% single support, 30% final double support) and the rest 20% swing phase. Figure 3c summarizes the overall steps analysis. The average stride lengths were 0.339 ± 0.106 and 0.389 ± 0.072 m for the left and right leg (*p* = 0.318, unpaired *t*-test). Average stride periods were 4.045 ± 0.723 and 4.213 ± 0.370 s for the left and right leg (*p* = 0.622, unpaired *t*-test). Similarly, average steps per minute (15.273 ± 2.967 and 14.322 ± 1.118 for the left and right legs) were not significantly different (*p* = 0.479, unpaired *t*-test). The over-ground walking speed of the study participant was 0.107 m/s.

### 3.3. Improvement of Forward Biking Ability with tES

For active cycling, a motorized bike with the option of both passive and active operation (MOTOmed viva2, RECK-Technik GmbH and Co. KG, Germany) was set at 10 cycles/min. The study participant was asked to try to exceed the speed while tES was delivered at 25 Hz. Over the course of the study, our participant showed a significant improvement in forward (*p* < 0.001; R2 value = 0.945) but not reverse biking speed, suggesting improved leg extensors function in the forward biking direction (Figure 3d). Although both forward and reverse biking could activate quadriceps, the sequence and extent of the muscle recruitment sequence might differ between the two types of biking, which might partly explain the differential findings. We also observed little or no difference in walking ability with and without the tES, suggesting a significant reorganization of spinal–supraspinal networks attributable to the repetitive exposure to spinal neuromodulation concomitant with exposure to a task-specific training paradigm as also observed recently with epidural stimulation [27].

### 3.4. Secondary Functional Improvements

The results reported by the participant in this study include descriptions of each of the functions noted above, plus noting improvements in sleeping patterns, with less insomnia and more deep sleep; the ability to perform exercises for longer periods and sitting posture also were improved. Throughout the research trial period, the study participant experienced a continuous improvement in the strength of the lower back and core muscles, which allowed her to sit up straighter and perform a variety of daily tasks. While engaging in standing exercises, the study participant gradually reduced her reliance on her hands for support, thus increasing the duration of standing solely on her feet. These enhancements have positive impacts on various aspects of her daily life. The increased lower body strength has helped the study participant to relieve some of the burden of the upper body and improved overall body balance. The participant noted, “Now I sit in a more upright posture and can work longer with less exhaustion”. These findings may result in a reduced cost of healthcare throughout life.

## 4. Discussion

Although researchers all over the world have searched for a cure for spinal cord injury, as yet, there is no known therapy to regenerate a damaged spinal cord [28]. Neuroregeneration, along with anti-inflammatory and preventive therapies, do not yet translate to humans with SCI [29]. In contrast, neurostimulation therapies can often be used for functional restoration and to minimize secondary conditions such as pain, respiratory, and cardiovascular functions, as well as improve gait performance [15,30,31].

Transcutaneous electrical stimulation (tES), a non-invasive method in which stimulating electrodes are placed on the skin to pass an electric current through the tissue underneath, has shown neuromodulatory effects on spinal cord neurocircuits [32]. We have recently shown that by combining locomotor training with tES at the lower thoracic (T11) and upper lumber (L1) spinal levels, an individual with over two decades of chronic paralysis suffering from Brown Séquard Syndrome from a motor-vehicle accident-induced cervical cord injury (C7), regained significant voluntary control on her pelvic limb [25]. In brief, after passing the clinical screening (Figure 1b, STAGE 1), 6 weeks of baseline test with tES were conducted to determine the best stimulation parameters for training (STAGE 2), followed by 16 weeks of training with tES to improve the lower extremity motor functions (STAGE 3). After training, the lower extremity motor score (LEMS) of the pelagic left leg, based on International Standards for Neurological Classification of SCI (ISNCSCI), increased significantly from 0 to 10 (*p* < 0.001; one-way repeated measures ANOVA; post hoc Tukey’s multiple comparison test) over the course of 16 weeks of tES and locomotor training. Further, after 6 weeks without stimulation or training (STAGE 4), the improved motor function did not change significantly (ISNCSCI score dropped from 10 to 8), thus sustaining an improved level of functional spinal–supraspinal connectivity. Hence, we further extended the study to examine if we can nurture additional neuronal plasticity and reinforce further motor learning. In particular, we further fine-tuned the stimulation parameters based on the lower extremity muscles’ responses to different functional tasks, including weight shifting during standing, reaching with the legs, squats, and reclined sit-ups (Figure 1b, STAGE 5). We found that in extensors-related activities such as standing, the participant responded well with 20 Hz stimulation; while attempting volitional effort during gait training, 25 Hz stimulation frequency was more beneficial along with the other fixed stimulation parameters (101 µsec biphasic pulses with 90 mA stimulation intensity). Stimulation details are shown in Figure 1a.

In a previous study, it was shown that multisite transcutaneous electrical stimulation along with locomotor training improves locomotor function in individuals with incomplete SCI [33]. However, no non-invasive treatment has yet shown restoration of over-ground ambulatory function in a wheelchair-bound individual with SCI. In the present study, the training protocol comprised 44 weeks of variable locomotor training (due to occasional restrictions for the COVID-19 pandemic) along with or without tES (STAGE 6). In the final assessments (STAGE 7), we found that even after this on-and-off training, our participant regained significant voluntary control over her lower extremities, and she could, for the first time, ambulate over-ground with the help of a walker (Pacer Gait Trainer, Rifton, USA) without any stimulation (Supplementary Video S1). Notably, the current study adopted the exercise progression principle in resistance training to improve muscle strength, corticospinal plasticity, and motor skill learning [34,35]. To the best of our knowledge, this is the first demonstration of walking restoration using a non-invasive treatment for an individual with severe chronic SCI.

The quantitative results demonstrating recovery of unassisted mobility over a period of 66 weeks in an individual who has been severely paralyzed and wheelchair-dependent for more than two decades using non-invasive spinal stimulation concomitant with task-specific training is of high significance. This demonstrates that the neuromuscular system is capable of adapting well beyond 6–12 months post-injury, a persistent long-term dogma that is rarely the case. Neuromodulation techniques have been used to successfully treat a variety of neurological conditions, such as spinal cord injury, stroke, multiple sclerosis, and children with cerebral palsy [16,22,36,37]. There is accumulating evidence that neuromodulation electrical modulation improves neuroregeneration and neural repair by affecting nervous system signals, which may help to enhance motor function and motor learning following neurological injury [38,39]. This can be accomplished by specifically controlling, suppressing, or increasing the activity of neurons and neural networks [40].

The success in the sensorimotor recovery of our participant sheds light on the future of non-invasive treatment for SCI paralysis. It also leaves an open question of whether non-invasive spinal cord neuromodulation can work similarly to or, in some individuals, even better than invasive epidural stimulation to restore lost functions, including voluntary movements, standing, over-ground walking, and sensation. If confirmed in future studies, tES could benefit a large population worldwide, to regain a significant level of function even after prolonged periods of paralysis.

We recognize the limitation necessary for interpreting the present data simply because it is a single case study. However, it should be pointed out that the strength of the present data, as in other case studies, by definition, has no valid control. These data do not address the issue of whether these observations imply that they represent some given population of specific subjects. The results are novel and demonstrate a level of plasticity in response to a novel combination of interventions, i.e., a neuromodulation procedure previously demonstrated consistently to transform the level of excitability and functionality of neural networks when combined with a specific series of activity-dependent interventions. The observations present functional levels that have not been previously reported using any intervention in a subject that has been paralyzed for a prolonged period. The present dogma is that such results are impossible, and the medical community routinely responds to patients accordingly. Thus, these results demonstrate what is possible with the new interventional strategy. It is also important because this result was obtained without being dependent on an extensive technological capability and, thus, has the potential of having a high impact in environments with limited medical technologies.

Despite the promising results, the current study had several limitations. Our findings may not be generalized to individuals with different levels or severity of SCI. Future trials with a larger sample size are warranted to validate the positive results in the present study. Furthermore, it remains unclear whether the stimulation location may have differential effects on the treatment outcomes. Future studies should determine the optimal electrode placement locations. Given that the mechanisms underlying the recovery remain unclear, future animal and human mechanistic studies should use advanced imaging technologies to explore the functional mechanisms of such recovery, which will help determine the optimal stimulation parameters for the best treatment outcomes in individuals with SCI.

## 5. Conclusions

This study shows for the first time how non-invasive spinal cord neuromodulation permanently restores volition control and over-ground ambulatory function in an individual with chronic paralysis. However, future studies are warranted to validate the results in more participants and to better understand the underlying mechanisms. Further explorations of the optimal stimulation parameters and their efficiency in more severely injured individuals are also needed.

## Data Availability

The data generated from this work can be obtained from the corresponding author upon request.

## Supplementary Materials

The following supporting information can be downloaded at https://www.mdpi.com/article/10.3390/jcm12237362/s1, Video S1: Study participant walks using a walking frame with AFOs (Ankle–Foot Orthoses) after completion of the study. No stimulation therapy was provided during or immediately before walking.
